# Draft Genome Sequence of Citrobacter freundii UFMG-H9, Isolated from Urine from a Healthy Bovine Heifer (Gyr Breed)

**DOI:** 10.1128/MRA.00387-20

**Published:** 2020-05-07

**Authors:** Silvia Giannattasio-Ferraz, Laura Maskeri, André P. Oliveira, Edel F. Barbosa-Stancioli, Catherine Putonti

**Affiliations:** a Departamento de Microbiologia, Instituto de Ciências Biológicas, Universidade Federal Minas Gerais, Belo Horizonte, MG, Brazil; b Bioinformatics Program, Loyola University Chicago, Chicago, Illinois, USA; c Empresa de Pesquisa Agropecuária de Minas Gerais–EPAMIG, Uberaba, MG, Brazil; d Department of Biology, Loyola University Chicago, Chicago, Illinois, USA; e Department of Computer Science, Loyola University Chicago, Chicago, Illinois, USA; f Department of Microbiology and Immunology, Stritch School of Medicine, Loyola University Chicago, Maywood, Illinois, USA; University of Maryland School of Medicine

## Abstract

Citrobacter freundii is a pathogen associated with antibiotic resistance and severe infections in humans. Here, we report the draft genome sequence of C. freundii strain UFMG-H9, an isolate from urine from a healthy Gyr heifer.

## ANNOUNCEMENT

Citrobacter freundii is a known human pathogen highly associated with the occurrence of bacteremia and antibiotic resistance (1). This species has been reported as a contaminant of wastewater (2) and food of animal origin such as milk and cheese (3). Strains of C. freundii have also been reported to cause urinary infections in humans (4). Here, we report the draft genome sequence of C. freundii strain UFMG-H9, isolated from urine from a healthy heifer belonging to a Gyr herd pure in origin, from the Agricultural Research Company of Minas Gerais State (EPAMIG) in Brazil.

Samples were collected in May 2019, and the methodology was previously approved by the Ethics Committee in Animal Experimentation of the Universidade Federal de Minas Gerais, Brazil (CEUA/UFMG; approval number 40/2019). All experiments were performed in accordance with relevant guidelines. To collect the urine, the vulva of the animal was washed with distilled water and soap, and then midstream urine was collected using a 50-ml sterile tube. The urine was kept at –20°C until processing (within 48 h). Aliquots of 2 ml were centrifuged. The supernatant was plated on lysogeny broth (LB) agar plates and incubated at 37°C overnight. Single colonies were picked, grown in LB at 37°C overnight, and then replated. This process of plating and liquid culture was repeated 3 times in order to obtain pure colonies. Following this, a single colony was inoculated in LB liquid medium and grown overnight with shaking at 37°C. A 1-ml aliquot was centrifuged, and the pellet was used for DNA extraction with the DNeasy UltraClean microbial kit (Qiagen, Hilden, Germany) according to the manufacturer’s instruction. The DNA was quantified using a Qubit fluorometer. The genus and species were determined prior to whole-genome sequencing by sequencing the 16S rRNA gene sequence. Next, the DNA was sent to the Microbial Genomic Sequencing Center (MiGS) at the University of Pittsburgh for library preparation and whole-genome sequencing. Briefly, the DNA was fragmented using an Illumina tagmentation enzyme, and indices were attached using PCR. The genome sequencing was conducted using the NextSeq 550 platform. Sequencing produced 2,854,920 pairs of 150-bp reads that were trimmed using Sickle v1.33 (https://github.com/najoshi/sickle). The genome was assembled using SPAdes v3.13.0 with the “only-assembler” option for k values of 55, 77, 99, and 127 (5). Then, the genome coverage was calculated using BBMap v38.47 (https://sourceforge.net/projects/bbmap/). Finally, the genome was annotated by the NCBI Prokaryotic Genome Annotation Pipeline (PGAP) v4.11 (6). Default parameters were used for all software tools unless otherwise noted.

C. freundii strain UFMG-H9 is 4,744,381 bp long with a GC content of 50.77%. The assembly includes 20 contigs with an *N*_50_ value of 525,854 bp, and the genome coverage is 164×. Annotation identified 4,315 coding genes and 75 tRNAs. Further analysis of this strain can increase our understanding of the urogenital tract of this species.

### Data availability.

This whole-genome sequencing (WGS) project has been deposited in GenBank under accession number JAAVMN000000000. The raw reads can be found in SRA under accession number SRR11455638. This sequencing is part of BioProject accession number PRJNA615899.
